# Sex-Dependent Responses to Maternal Exposure to PM_2.5_ in the Offspring

**DOI:** 10.3390/antiox11112255

**Published:** 2022-11-15

**Authors:** Hui Chen, David Van Reyk, Annabel Oliveira, Yik Lung Chan, Stephanie EL Town, Benjamin Rayner, Carol A Pollock, Sonia Saad, Jacob George, Matthew P Padula, Brian G Oliver

**Affiliations:** 1 School of Life Sciences, Faculty of Science, University of Technology Sydney, Sydney, NSW 2007, Australia; 2 Respiratory Cellular and Molecular Biology, Woolcock Institute of Medical Research, The University of Sydney, Sydney, NSW 2037, Australia; 3 Children’s Cancer Institute, UNSW Sydney, Sydney, NSW 2052, Australia; 4 Kolling Institute of Medical Research, Royal North Shore Hospital, The University of Sydney, Sydney, NSW 2065, Australia; 5 Storr Liver Centre, The Westmead Institute for Medical Research, Westmead Hospital and The University of Sydney, Sydney, NSW 2037, Australia

**Keywords:** particulate matter, inflammation, ER stress, oxidative stress, fibrosis, liver metabolic profile

## Abstract

**Objective**: Particulate matter (PM) with a diameter of 2.5 μm or less (PM_2.5_) can cross the blood-placental barrier causing adverse foetal outcomes. However, the impact of maternal exposure to low-levels of PM_2.5_ on liver health and the metabolic profile is unclear. This study aimed to investigate hepatic responses to long-term gestational low-dose PM_2.5_ exposure, and whether the removal of PM after conception can prevent such effects. **Method**: Female Balb/c mice (8 weeks) were exposed to PM_2.5_ (5 μg/day) for 6 weeks prior to mating, during gestation and lactation to model living in a polluted environment (PM group). In a sub-group, PM_2.5_ exposure was stopped post-conception to model mothers moving to areas with clean air (pre-gestation, Pre) group. Livers were studied in 13-week old offspring. **Results**: Female offspring in both PM and Pre groups had increased liver triglyceride and glycogen levels, glucose intolerance, but reduced serum insulin and insulin resistance. Male offspring from only the Pre group had increased liver and serum triglycerides, increased liver glycogen, glucose intolerance and higher fasting glucose level. Markers of oxidative stress and inflammation were increased in females from PM and Pre groups. There was also a significant sex difference in the hepatic response to PM_2.5_ with differential changes in several metabolic markers identified by proteomic analysis. **Conclusions**: Maternal PM exposure exerted sex-dependent effects on liver health with more severe impacts on females. The removal of PM_2.5_ during gestation provided limited protection in the offspring’s metabolism regardless of sex.

## 1. Introduction

Air pollution is ranked 9th as the leading global cause of disease burden [1]. Air pollution is comprised of gas and. Particulate matter (PM) is the particle component suspended in polluted air, including both solid and liquid particles, which can contain carbon, sulphates, nitrate, and toxic heavy metals [2,3,4,5]. About 91% of the world’s population breathe in air with a quality below WHO standard [4,6]. The latest Lancet Commission on pollution and health reported 4.9 million premature deaths worldwide due to air pollution in 2019, affecting more people than any other pollutant. This has driven the World Health Organisation (WHO) to lower the guideline on the safe PM_2·5_ exposure limits from 10 μg/m^3^ to 5 μg/m^3^ [7].

Although 90% of people whose health is harmed by air pollution live in developing countries [6,8], in places where air quality is relatively good, such as Australia, the residents are still affected by the effects of PM exposure, primarily due to traffic-related air pollution (TRAP) [4]. People who reside 50 to 500 m or less from major roadways are more likely to experience chronic low-level TRAP exposure and the resulting negative health impacts [9,10,11,12,13]. While the gaseous component of TRAP dissipates quickly, PM remains airborne for a long period of time. PM smaller than 10 μm (PM_10_) is respirable, and PM_2.5_ can reach the alveoli. PM_2.5_ can enter the systemic circulation leading to a range of adverse health effects such as asthma, cardiopulmonary disease, and adverse birth outcomes [9,10,11,12,13]. An association between direct exposure to high levels of PM_2.5_ and metabolic disorders, including metabolic dysfunction related to fatty liver changes, have also been suggested [2,14,15,16,17]. However, the effect of exposure which occurs during pregnancy on the offspring is not known,

PM_2.5_ crosses the blood-placental barrier and circulates in foetal blood, and thus is considered an in utero environmental toxin [18]. Population research in areas with heavy PM_2.5_ pollution showed that in utero PM exposure can lead to miscarriage, intrauterine growth restriction, preterm birth, and low birth weight [19,20]. As foetal development is a crucial window in which the likelihood of developing various chronic diseases in adulthood can be modified, in utero PM exposure may affect foetal organ development and raise the offspring’s vulnerability to non-communicable diseases later in life [18,19,20]. Most of the research on in utero effects of PM_2.5_ has focused on cardiopulmonary outcomes [18,21].

The liver is a key metabolic organ that governs and maintains energy metabolic homeostasis for both lipids and glucose [22,23]. Disorders of hepatic metabolism can cause the development of liver diseases, such as nutrient metabolic dysfunction associated with fatty liver disease (MAFLD) due to de novo lipid synthesis, and type 2 diabetes mellitus from insulin resistance and dysregulated gluconeogenesis [22,23]. Human studies have suggested a close link between air pollution and the development of hepatic steatosis and fibrosis, including cirrhosis [16,24,25]. However, little is known about the impact of maternal PM_2.5_ exposure on hepatic outcomes in the offspring. This can be challenging to study in humans, where it is unlikely that we can separate in utero from postnatal exposure. Thus, it is difficult to determine whether the changes in the liver were programmed by maternal exposure [17,26,27].

Several animal models have shown the involvement of PM_2.5_ and black carbon in inducing liver inflammation, endoplasmic reticulum (ER) stress, oxidative stress, and fibrotic changes [28,29,30]. There are some similarities in the birth outcomes between PM_2.5_ and another common intrauterine environmental toxin, cigarette smoke, including intrauterine underdevelopment and low birth weight [18,31], which are known risk factors for abnormal liver development and glucose intolerance [32]. Indeed, maternal smoking can change liver glucose and lipid metabolism resulting in glucose intolerance and increase in triglycerides in both liver and blood in adulthood in both sexes, driven by increased inflammatory responses, oxidative stress, and deregulation of lipid metabolic markers in both sexes [31,33]. However, whether maternal PM_2.5_ inhalation during pregnancy can also affect liver function in the offspring is unknown, neither is knowledge of any sex differences. High levels of PM_2.5_, as seen in heavily populated regions such as India and Mexico, have been commonly used in experimental models, whereas low levels of PM_2.5_ considered to be “safe” have rarely been studied. We showed that even in utero exposure to “safe” levels can be detrimental to lung health, also driven by increased inflammatory responses and oxidative stress [34]. Therefore, we hypothesised that in utero exposure to a low level of PM_2.5_ may also cause heightened responses in inflammation, ER stress, oxidative stress, and fibrotic changes in the liver, serving as the mechanism underlying abnormal lipid and glucose metabolic profile in offspring of both sexes. To model the effect of moving to an area with cleaner air, PM_2.5_ exposure was stopped post conception allowing us to determine the future risk of metabolic disorders in the offspring. In offspring, we measured markers of inflammation, ER stress, oxidative stress, fibrosis, and lipid deposition in the livers of 13-week old (adult) offspring of both sexes.

## 2. Materials and Methods

### 2.1. Animal Model

**Figure 1:**
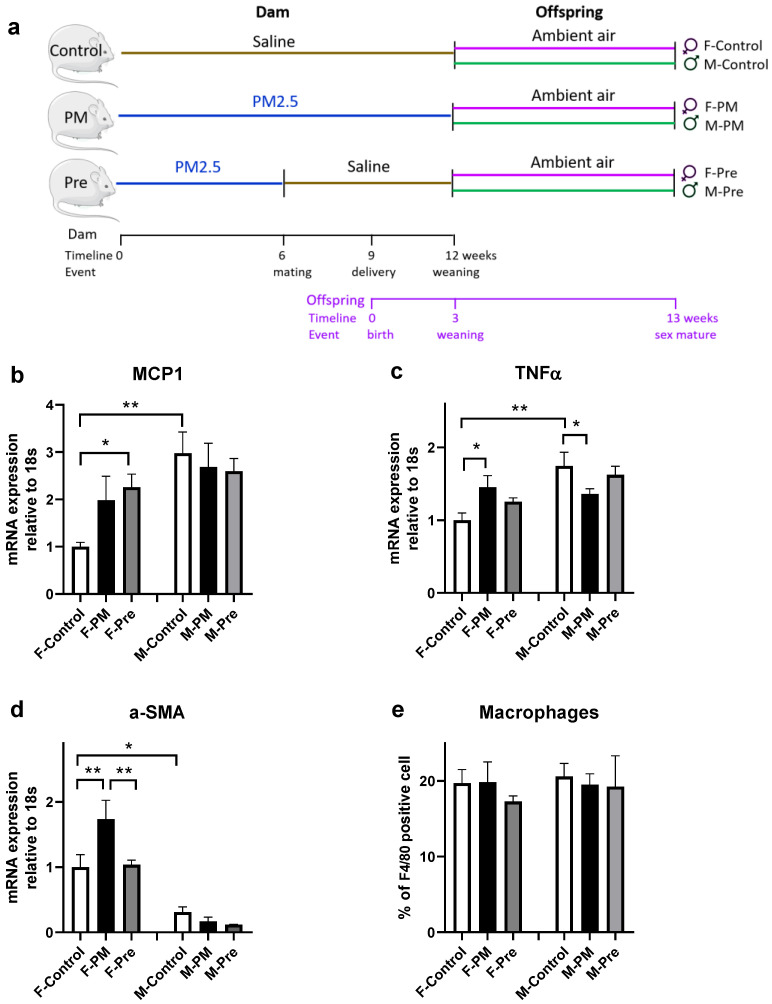
**Schematic of the Experimental Model and Characterisation of inflammatory Markers in Female and Male Offspring.** Experimental design (**a**). mRNA levels of MCP1: monocyte chemoattractant protein 1 (**b**), TNFα: tumour necrosis factor α (**c**), and α-SMA: α-smooth muscle actin 2 (**d**), as well as F4/80 positive macrophages (**e**). Results are expressed as mean ± SEM, n = 6 (**a**–**c**), n = 3 (**d**), n = 3–4 (**e**). * *p* < 0.05, ). ** *p* < 0.01. F: female; M: male; PM: offspring from dams continuously exposed to PM2.5; Pre: offspring from dams exposed to PM2.5 before conception only.

The animal study was approved by the Animal Care and Ethics Committee at the University of Technology Sydney (ETH17-1998) and followed the Australian National Health and Medical Research Council Guide for the care and use of laboratory animals. The animal model has been previously published, which reported lung pathology in the same cohort [34]. All mice were housed in individually ventilated cages (20 ± 2 °C, 12 h light, 12 h dark cycle, lights on at 06:00 h) with unlimited access to standard laboratory rodent chow and drinking water in a PC2 facility. Female Balb/c mice (8 weeks, Animal Resource Centre, WA, Australia) were separated into 3 groups (Figure 1a). PM_2.5_ was collected on a busy roadside in Hong Kong for 18 days during summer by the URG PM samplers (URG-2000-30EH, 8 L/min) using a 47 mm Teflon (Pall Life Sciences, Ann Arbor, MI, USA) and quartz-fiber filters (Whatman, Clifton, NJ, USA). Briefly, two groups were given roadside PM_2.5_ (5 μg suspended in 40 μL saline, nasal instillation, once daily) 6 weeks before mating to model mothers living in a mildly polluted environment (e.g., along a busy road), whilst the third group was the control group (40 μL of saline, delivered nasally, Control). These treatments in the dams continued during pregnancy and lactation. However, a sub-group of PM_2.5_ exposed dams was switched to saline during pregnancy and lactation (Pre). The pups were not subjected to direct PM_2.5_ exposure.

At 12 weeks postpartum, male and female offspring underwent an intraperitoneal glucose tolerance test following our published protocol [35]. An area under the curve (AUC) of the glucose change during the test was calculated for each mouse. At 13 weeks, overnight fasted offspring were harvested after deep anaesthesia (isoflurane 2%), and the body weight was measured. Blood was collected via cardiac puncture, and serum was kept to measure insulin levels using a commercial ELISA kit (Abnova, Taipei, Taiwan) as per the manufacturer’s instructions. The formula: insulin (μU/mL) × glucose (mM)/22.5 was used to calculate the Homeostatic Model Assessment of Insulin Resistance (HOMA-IR) index. Blood triglyceride levels were measured by an in-house method using glycerol standards (Sigma-Aldrich, St. Louis, MO, USA) and triacylglycerol reagent (Roche Diagnostics, Basel, Switzerland), as we have published previously [36,37].

The livers from both sexes were collected, weighed, and then fixed in 4% *w*/*v* formalin for histology, or snap-frozen and stored at −80 °C for protein and mRNA measurements. Both female and male offspring were included in determining sex differences in response to maternal PM_2.5_ exposure.

### 2.2. Immunohistochemistry

Paraffin-embedded livers were sectioned (5 μm), deparaffinised in xylene, and hydrated in a decreasing gradient of ethanol. The antigens were then retrieved by microwaving on high (900 W, 15 min) in EDTA/Citrate Buffer (1 mM EDTA, pH 8.0). Then, the liver tissue sections were washed and incubated with Peroxidase Block (Dako Agilent, Santa Clara, CA, USA). After blocking, the sections were incubated with the peroxidase-conjugated primary antibody (F4/80, 1:200 females, 1:100 males, Novus Biological, CO, USA) for 1 h, followed by the secondary antibody (anti-rabbit HRP, 1:200, Dako, CA, USA) for 40 min, followed by PBST washes. The colour was developed using diaminobenzidine (DAB) chromogen (Dako, Agilent, Santa Clara, CA, USA). Cell nuclei and cytoplasm were stained with Haematoxylin and Eosin. 

Periodic acid–Schiff (PAS) was used to detect polysaccharides suggestive of glycogen storage and function [38]. The sections were then incubated in 1% *w/v* periodic acid, followed by Schiff’s reagent (15 min), Mayer’s haematoxylin, and finally Scott’s blue. Then, the sections were dehydrated in increasing grades of ethanol (1 × 95%, 2 × 100%) and xylene before cover-slipped.

Picrosirius red (PSR) staining allows visualising collagen deposition [39]. The liver sections were incubated with PSR solution (Sigma-Aldrich, MO, USA), then rinsed in 0.5% *v/v* glacial acetic acid, and dehydrated through increasing grades of ethanol and xylene prior to mounting and cover-slipping. The sections were quantified with ImageJ (National Institutes of Health, Bethesda, MD, USA).

### 2.3. Tissue Lipid Quantification

Oil Red O (ORO) staining was used to quantify the lipids in frozen liver tissue as we have previously published [35]. ORO working solution (300 μL, ORO stock solution (0.25% (wt/vol), Merck, NJ, USA): 10% dextran at 6:4) was added to the frozen liver pieces (30–40 mg pieces) and homogenised (6500 g, 18 s × 2, 30 s pause, Precellys^TM^ Tissue Homogeniser, Bertin Technologies, IDF, France). The samples were then rinsed with 60% isopropanol and vortexed for 5 min. The samples were then centrifuged (13,000 rpm, 2 min) with the supernatant removed and rinsed again with 60% isopropanol. Isopropanol was added to the pellet and centrifuged (14,000 rpm, 5 min), collecting the supernatant. This isopropanol process was repeated twice more (using 750 μL and then 500 μL, isopropanol) and centrifuged (14,000 rpm, 5 min) to remove any tissue fragments. The samples were transferred to a 96-well plate for colour detection (520 nm, Tecan Infinite^®^ M1000, ZH, Switzerland). The readings obtained were then corrected for tissue weight (mg).

### 2.4. Real-Time PCR

Total mRNA was extracted from frozen liver tissue using TriZol reagent (Life Technologies, CA, USA) and first-strand cDNA was generated using M-MLV Reverse Transcriptase, RNase H, Point Mutant Kit (Promega, Madison, WI, USA). The mRNA expression of target genes involved in inflammation, oxidative stress and fibrosis was quantified with primers and probes pre-validated and optimised by the manufacturer (Table 1, Thermo Fisher, Waltham, CA, USA) and standardised to the housekeeping gene 18s RNA. The probes of the target genes were labelled with FAM^®^ or SYBR^®^, and those for 18s RNA were labelled with VIC^®^. The average mRNA expression of the target gene in the F-Control group was used as the calibrator, against which all other groups were expressed as fold changes to the F-Control group.

### 2.5. Western Blotting

Frozen liver tissue (10–100 mg) was homogenised in 200 μL of cold lysis buffer and 2X Laemmli buffer using Precellys^TM^ Tissue Homogeniser (6500 g, 18 s × 2, Bertin Technologies, IDF, France). The homogenates were centrifuged (1500× *g*, 5 min, 4 °C). NuPage Novex 4–12% Bis–Tris gels were used to separate the protein samples (Life Technologies, Carlsbad, CA, USA), which were then transferred to PVDF membranes (Pierce, Rockford, IL, USA). A 5% skim milk solution was used to block the membranes before the incubation with primary antibodies of markers for oxidative stress and ER stress (manganese superoxide dismutase (MnSOD, 1:0000, Millipore, MA, USA), heat shock protein 90 (HSP90, 1:1000, Cell Signalling Technology, MA, USA); X-box binding protein 1-spliced (XBP-1s, 1:1500, Bio legend, CA, USA); C/EBP homologous protein (CHOP, 1:1000, Novus Biological, CO, USA); β-Actin (Bio-Rad Laboratories, CA, USA) 1:3000) followed by horseradish peroxidase-conjugated secondary antibody (1:3000, Abcam, Cambridge, UK). SuperSignal West Pico Chemiluminescent substrate and Fujifilm LAS-3000 (Fujifilm, Tokyo, Japan) were used to detect the protein bands (Thermo Fisher Scientific, CA, USA). The band densities were quantified with ImageJ (National Institutes of Health, Bethesda, MD, USA). The housekeeping protein β-actin was used as the calibrator for ratio calculation.

### 2.6. LC/MS/MS

Protein and metabolic pathways were examined using proteomic techniques. Samples (5 µL) were loaded onto a nanoEase Symmetry C18 trapping column (180 m × 20 mm) (3 min/15 L/min) via an Acquity M-class nanoLC system (Waters, Milford, MA, USA), and then washed onto a PicoFrit column (75 mID × 350 mm; New Objective, Woburn, MA, USA) packed with SP-120-1.7-ODS-BIO resin. Eluted peptides were loaded into the source of a Q Exactive Plus mass-spectrometer (Thermo Scientific) using 5–30% MS buffer B (98% Acetonitrile + 0.2% Formic Acid) − 90 min, 30–80% MS buffer B − 3 min, 80% MS buffer B (2 min), 80–5% for 3 min. Eluted peptides were ionised at 2400 V. A data-dependent MS/MS (dd-MS2) experiment was carried out using a survey scan of 350–1500 Da at 70,000 resolution for peptides of charge state 2+ or greater with an AGC target of 3e6 and maximum Injection Time of 50 ms. Peptides (top 12) were fragmented in the HCD cell using an isolation window of 1.4 m/z, an AGC target of 1 × 10^5^ and a maximum injection time of 100 ms. These were analysed in the Orbitrap analyser at 17,500 resolution; production fragment masses were measured (mass-range of 120–2000 Da). The mass of the precursor peptide was then excluded for 30 s.

MS/MS datafiles analysed using Peaks Studio X Pro (mouse proteome from UniProt) with a database of common contaminants with the following parameter settings. Fixed modifications: none. Variable modifications: propionamide, oxidised methionine, deamidated asparagine. Enzyme: semi-trypsin. The number of allowed missed cleavages: 3. Peptide mass tolerance: 10 ppm. MS/MS mass tolerance: 0.05 Da. Searchers were refined to include peptides with a –log10P score characterised by a False Discovery Rate (FDR) of <1%. The score is that where decoy database search matches were <1% of the total matches. Label-Free Quantification (LFQ) was performed using the PEAKS Q module. The protein’s gene name and fold changes were submitted to StringDB (https://string-db.org/ (accessed on 5 November 2022)) for Functional Enrichment using the “Proteins with Ranks/Values” Search.

### 2.7. Statistical Analysis

The results are expressed as mean ± standard error of the mean. Data were analysed by two-way ANOVA with Tukey post hoc tests to examine the differences between sexes and maternal groups (GraphPad Prism 8). For individual MS/MS data sets, male and female results were analysed separately with one-way ANOVA with Tukey post hoc tests. *p* < 0.05 was considered statistically significant.

## 3. Results

### 3.1. Effect of Maternal PM_2.5_ Exposure on Females

Female PM offspring had significantly smaller body weight than the Control at adulthood (*p* < 0.05, Table 2), without any difference in liver weight. Female PM offspring had a significantly greater triglyceride concentration in the liver and marked glucose intolerance during IPGTT (both *p* < 0.05 F-PM vs. F-Control, Table 2). Blood triglyceride level in the F-PM group was non-significantly reduced by 13% (Table 2). Glycogen storage reflected by PAS staining was significantly increased in F-PM mice compared to the Controls (*p* < 0.05 F-PM vs. F-Control, Table 2). However, they had lower fasting insulin levels (*p* < 0.05 F-PM vs. F-Control), while insulin resistance was also lower than in the Control females reflected by lower HOMA values (*p* < 0.01, Table 2).

**Figure 2:**
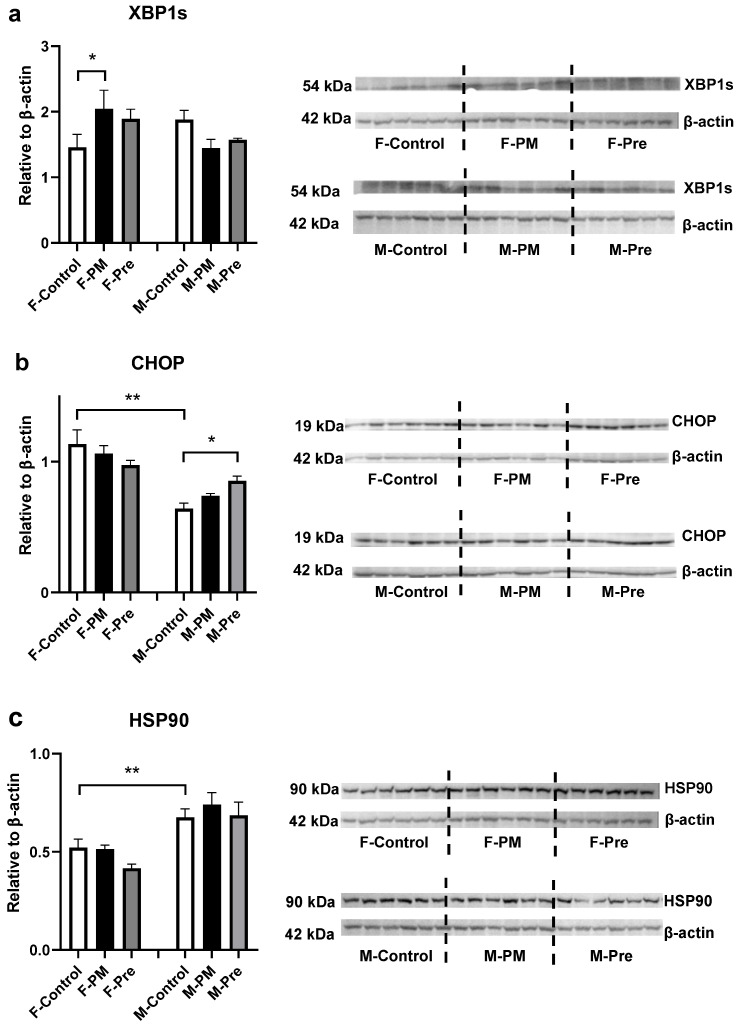
**ER Stress Markers in Female and Male Offspring.** Protein expression levels of XBP1s (**a**), CHOP (**b**), and HSP90 (**c**). Results are expressed as mean ± SEM, *n* = 6. * *p* < 0.05, ** *p* < 0.01. CHOP: C/EBP homologous protein, HSP90: heat shock protein 90, XBP1s: X-box binding protein 1 spliced. PM: offspring from dams continuously exposed to PM2.5; Pre: offspring from dams exposed to PM2.5 before conception only.

**Figure 3:**
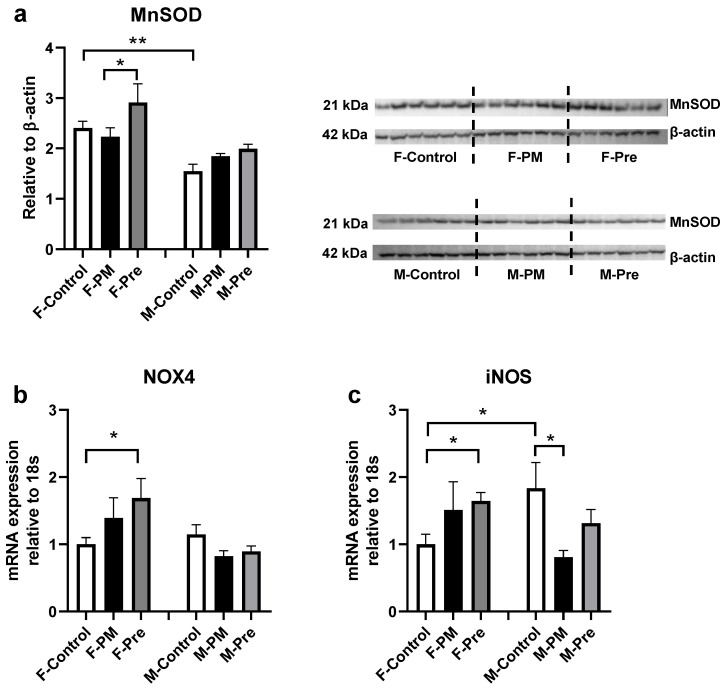
**Oxidative Stress Markers in Female and Male Offspring.** Protein expression levels of MnSOD (**a**) and mRNA expression levels of iNOS (**b**) and NOX4 (**c**). Results are expressed as mean ± SEM, *n* = 6. * *p* < 0.05; ** *p* < 0.01. iNOS: inducible nitric oxide synthase, MnSOD: manganese superoxide dismutase, NOX4: NADPH oxidase 4. PM: offspring from dams continuously exposed to PM2.5; Pre: offspring from dams exposed to PM2.5 before conception only.

**Figure 4:**
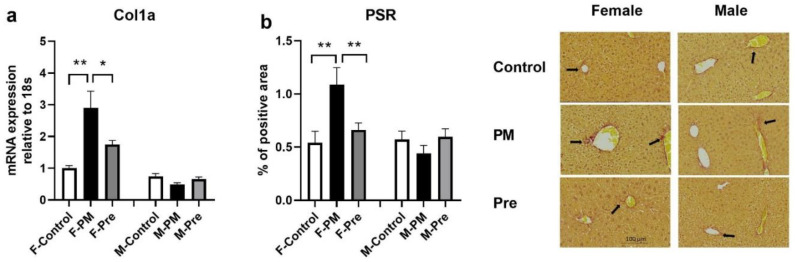
**Fibrosis Markers in Female and Male Offspring.** mRNA expression level of collagen Iα1 (ColIα1) (**a**), the relative density of picrosirius red (PSR)-positive area and representative images of PSR staining (indicated by black arrows) (**b**). Results are expressed as mean ± SEM, *n* = 6 (**a**), *n* = 5 (**b**). * *p* < 0.05, ** *p* < 0.01. PM: offspring from dams continuously exposed to PM2.5; Pre: offspring from dams exposed to PM2.5 before conception only.

Regarding inflammatory markers in the liver, in utero PM_2.5_ exposure doubled MCP1 mRNA expression (Figure 1b) and significantly increased the expression of TNFα (*p* < 0.05 F-PM vs. F-Control, Figure 1c, *p* < 0.05 F-PM vs. F-Control) and α-SMA (Figure 1d), without any impact on active inflammatory macrophage number (Figure 1e). The ER stress marker XBP1s was significantly higher in F-PM mice (*p* < 0.05 vs. F-Control, Figure 2a); no change was found for the other two ER stress markers CHOP and HSP90 (Figure 2b,c). Liver oxidative stress markers, MnSOD, NOX4 and iNOS were not significantly increased by maternal PM exposure (Figure 3). However, liver Col1a mRNA expression (*p* < 0.01 F-PM vs. F-Control, Figure 4a) and PSR (*p* < 0.01 F-PM vs. F-Control, Figure 4b) content were significantly increased in F-PM mice compared to female Controls. PSR staining was visible mainly around blood vessels.

### 3.2. Effect of PM_2.5_ Removal Post Conception in Female Offspring

PM_2.5_ removal during pregnancy partially ameliorated the reduced body weight in female offspring resulting from continuous PM_2.5_ exposure (pregnancy to lactation) (Table 2). However, more triglyceride accumulated in the livers of Pre-exposed female offspring (*p* < 0.01 vs. F-Control), whereas blood triglyceride level was reduced in these mice (*p* < 0.05 vs. F-Control, Table 2). In addition, the removal of PM_2.5_ post conception did not change glucose tolerance, insulin levels or HOMA index, but further reduced the fasting blood glucose.

PM_2.5_ removal during pregnancy did not prevent the changes in MCP1 and TNFα expression in F-PM mice (Figure 1a,b), but normalised α-SMA level (*p* < 0.01 F-Pre vs. F-PM, Figure 1c). This intervention did not affect ER stress markers (Figure 2), but increased all oxidative stress markers measured in this study, including MnSOD, NOX4, and iNOS (all *p* < 0.05, Figure 3). It also prevented the increase in Col1a and PSR levels (both *p* < 0.01 F-Pre vs. F-PM, Figure 4).

### 3.3. Effect of Maternal PM_2.5_ Exposure on Males

Maternal PM_2.5_ exposure did not affect male offspring’s body weight, liver weight, nor lipid concentration in the blood and liver. However, it significantly increased fasting glucose (*p* < 0.05 vs. Male Control) without affecting glucose tolerance during IPGTT and the insulin resistance index HOMA (Table 2).

In utero PM_2.5_ exposure suppressed TNFα expression (*p* < 0.05, Figure 1b), without affecting other inflammatory markers measured (Figure 1), nor any of the ER stress markers, namely XBP1s, CHOP, and HSP90 (Figure 2). However, the oxidative stress marker iNOS was significantly lower in M-PM mice than in M-Control mice (*p* < 0.05, Figure 3c). There was no significant change in Col1a and PSR levels in M-PM group (Figure 4).

### 3.4. Effect of PM_2.5_ Removal Post Conception in Male Offspring

PM_2.5_ removal during post conception did not affect body weight, but reduced liver weight (*p* < 0.01 vs. male Control), and serum triglyceride (*p* < 0.05 vs. male Control), but increased liver triglyceride concentration (*p* < 0.01 vs. both male Control and PM, Table 2). Liver glycogen content shown by PAS staining was also increased (*p* < 0.01 M-Pre vs. M-Control, Table 2). In addition, PM_2.5_ removal post conception caused significant glucose intolerance (*p* < 0.01 vs. both M-Control and M-PM) and increased fasting glucose level by 19% (*p* < 0.01 vs. M-Control, Table 2). However, the insulin resistance index HOMA was not changed, possibly due to increased endogenous glucose production.

There was no impact on liver inflammatory markers by removing PM_2.5_ after post conception (Figure 1). However, the ER stress marker CHOP was significantly higher in M-Pre mice (*p* < 0.05 vs. M-Control, Figure 2b). PM removal during pregnancy did not impact the oxidative stress markers (Figure 3), nor affected Col1a and PSR levels in M-Pre group (Figure 4).

### 3.5. Sex Differences

Control males have higher liver levels of MCP1 and TNFα than female littermates (both *p* < 0.01 F-Control vs. M-Control, Figure 1a,b). However, α-SMA was lower in males than in female littermates (*p* < 0.05 F-Control vs. M-Control, Figure 1c). The ER stress markers showed a differential sex difference between control females and males, with lower CHOP and higher HSP90 protein levels in males compared with female littermates (both *p* < 0.01, F-Control vs. M-Control, Figure 2b,c). Endogenous antioxidant MnSOD was lower (*p* < 0.01 F-Control vs. M-Control, Figure 3a), whereas the oxidative stress marker iNOS was higher in the male Control mice compared with their female littermates (*p* < 0.05 F-Control vs. M-Control, Figure 3c).

**Figure 5:**
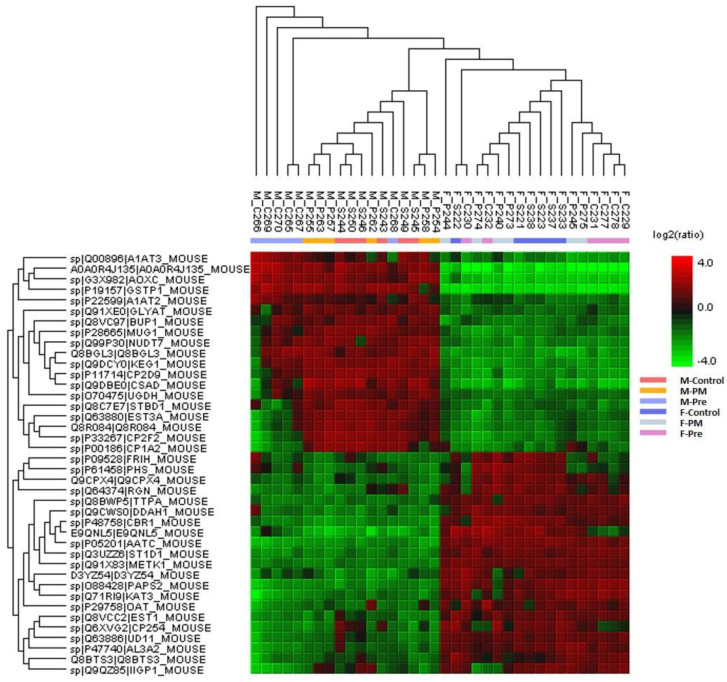
**Protein profile heatmap from Peak Studio XPro.** Cell colour represents the log2(ratio) to the average abundance across different samples, highlighting the distinct sex-specific differences in protein abundance. n = 6 for each group. PM: offspring from dams continuously exposed to PM_2.5_; Pre: offspring from dams exposed to PM2.5 before conception only.

**Figure 6:**
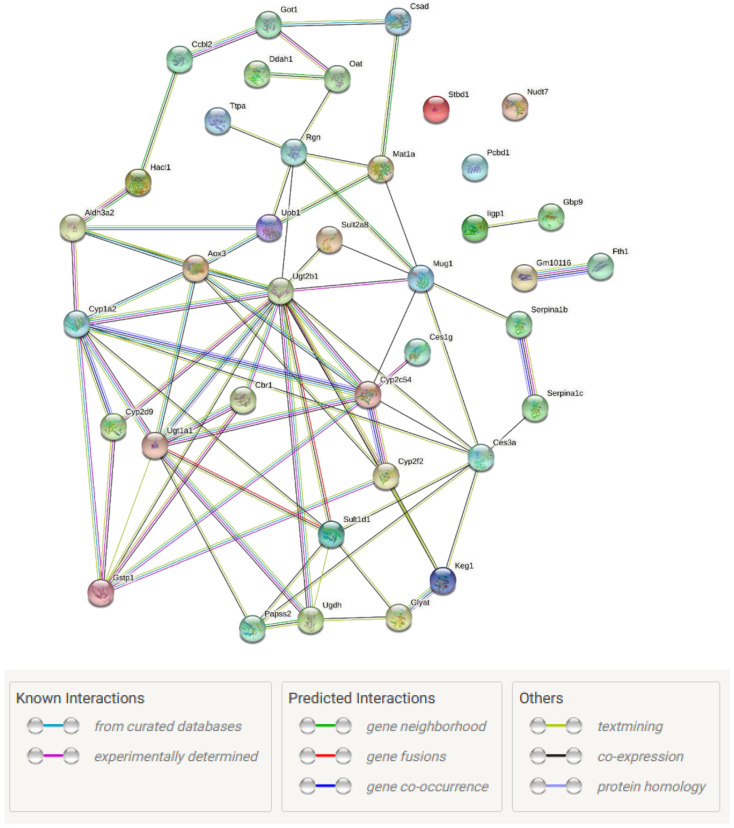
Protein interaction network generated by String-DB analysis of the differentially abundant proteins listed in Figure 5, highlighting the interconnectedness of these sex-specific proteins. Colours indicate the following.

The most marked sex difference lies in protein abundance in the liver, as shown in Figure 5, where differences in abundance are segregated in a sex-specific manner. Pathway analysis using https://string-db.org (accessed on 26 July 2021) (Figure 6) demonstrates the interconnection of the differentially abundant proteins with the proteins at the centre of the network that connect to different pathways being UDP glucuronosyltransferase 2 family polypeptide B1 (Ugt2b1) and Cytochrome P450 family members, which regulate drug and lipid metabolism [40]. Those identified within this network are involved in these sex-dependent responses to affect processes of nutrient oxidation, detoxification, glucose and lipid metabolism, mitochondria, and antioxidant synthesis (Table 3, negatively changed proteins shown in Supplementary Table S1). For example, the abundance of Cytochrome P450 1A2 (Cypla2, oxidising), Starch Binding Domain 1 (Stbd1, eliminating glycogen), Cytochrome P450, family 2, subfamily f, polypeptide 2 (Cyp2f2, detoxification and lipase), and Carboxylesterase (Ces)1/Ces3a (detoxification, lipid metabolism) were higher in males; whereas the abundance of the other proteins (Table 3), which are mostly involved in nutrient metabolism, was all higher in the female littermates. The enriched functional categories are listed in Supplementary Table S2, which confirmed the change of markers involved in nutrition metabolism that led to glucose and lipid metabolic disorders by maternal PM_2.5_ exposure.

When each sex was investigated alone, more proteins were changed in the males than in their female littermates, especially in the M-Pre group. These proteins and their functions are shown in Table 3. Ornithine Aminotransferase (Oat) was increased, and Carbonyl reductase (Cbr)1 was reduced in the M-PM offspring (*p* < 0.05 M-PM vs. M-Control), both of which were close to the values in control mice in the M-Pre group (*p* < 0.01 M-PM vs. M-Pre). Regucalcin (Rgn) was reduced in the M-PM offspring (*p* < 0.05 vs. M-Control), which was not changed in the M-Pre group. Some proteins were only changed in the M-Pre group. Dimethylarginine Dimethylaminohydrolase (Ddah)1 was increased (*p* < 0.05 vs. M-Control) while Long-chain-aldehyde dehydrogenase (Aldh3a2, *p* < 0.01 vs. M-Control), Glutamic-Oxaloacetic Transaminase 1 (Got1, *p* < 0.01 vs. M-Control), and Nudt7 (*p* < 0.05 vs. M-PM group) were reduced in the M-Pre group. Seven proteins (Stbd1, Cyp1a2, UDP-glucuronosyltransferases (Ugt)2b1, Ces1, Ces3a, Cyp2f2, Interferon-Inducible GTPase (Iigp)1) in the M-Pre group were significantly lower than those in both M-Control and M-PM groups (*p* < 0.05, Table 3). In female offspring, Ddah1 was increased, while Stbd1, Ces1 and Iigp1were reduced in F-PM group (*p* < 0.05 vs. F-Control), these were not different in the F-PM group. In the F-Pre group, Cbr1 and Stbd1 were significantly reduced, while Sult1a1 was significantly increased (*p* < 0.05 vs. F-Control, Table 3).

## 4. Discussion

Sexual dimorphism in disease susceptibility is a naturally occurring phenomenon. In general, women seem to be protected from most metabolic disorders, especially before menopause, such as dyslipidemia and glucose intolerance that render individuals at high risk of cardiovascular diseases and type 2 diabetes [41,42]. Consistently, in this study, control male offspring had higher levels of inflammatory and oxidative stress markers than female offspring. However, against these unfavourable baseline sexual biases and our hypothesis, in response to low level maternal PM_2.5_ exposure, females were more prone to liver pathology and lipid disorders, whereas only some inflammatory responses and collagen deposition were prevented by removing PM_2.5_ during gestation in female offspring. Proteomic analysis suggested that the removal of PM_2.5_ only during pregnancy may not be sufficient to completely prevent the adverse impacts of PM exposure in the dams prior to pregnancy.

The liver maintains metabolic homeostasis where the blood from the digestive system drains into the liver via the hepatic portal vein, which metabolises and stores dietary-derived carbohydrates, lipids, and proteins [22]. Excess blood glucose is stored as glycogen and triglycerides via glycolysis and Krebs cycle; liver gluconeogenesis from free fatty acids and amino acids occurs when the body is in need of more glucose [22,23]. In this study, blood glucose levels were higher, as reflected in the AUC value of the glucose tolerance tests in F-PM mice. However, low HOMA index and fasting serum insulin levels in these mice suggest relative insulin insufficiency in PM-female offspring. This may cause delayed glucose clearance/uptake during the glucose tolerance test in these mice. Indeed, a previous study using diesel exhaust PM_2.5_ showed that perinatal exposure resulted in β-cell dysfunction [43]. The situation is similar to maternal smoking where fasting insulin levels are reduced in the offspring [35]. Additionally, liver triglyceride and glycogen concentrations were both increased, suggesting that extra glucose is stored in the liver, instead of being used to generate ATP. Stbd1, involved in the breakdown of glycogen, was reduced in F-PM offspring, which may explain increased liver glycogen storage. The inhibition of carboxylesterase family member Ces1 impairs liver triglyceride lipolysis, further leading to increased triglyceride content in the F-PM liver [44,45]. Removing PM_2.5_ in dams from conception did not impact glucose intolerance and triglyceride concentration in the liver, but reduced fasting glucose and circulating triglyceride levels.

PM_2.5_ is a known oxidant, increasing systemic ROS production [46]. Overproduced ROS from the precursor NOX4 or iNOS can overwhelm endogenous antioxidants leading to oxidative damage [47]. In this study, oxidative stress markers were increased in female PM offspring, which was partially ameliorated by PM removal during the intrauterine period. The oxidative stress response in the F-PM mice activated another two mechanisms, inflammatory responses and ER stress. Previous studies using high doses of PM_2.5_ exposure showed Kupffer cell (endogenous liver macrophages) driven inflammatory responses reflected by increased cell number and related cytokines such as TNFα and MCP1 [4,5,8,29,48,49]. Enhanced local inflammation is then closely related to fibrosis [5]. Indeed, PM_2.5_ exposure has been shown to increase the fibrotic marker ColIα1 [50,51,52]. It can also disturb liver homeostasis and increase ROS production in hepatic stellate cells in vitro [14]. Stellate cells surrounding hepatic sinusoids differentiate into myofibroblasts which express α-smooth muscle actin and collagen I, and plays a vital role in fibrosis by synthesis of collagen and other extracellular matrix constituents [53,54]. In this study, maternal PM_2.5_ exposure resulted in increased stellate cells in F-PM offspring’s liver, with increased inflammatory responses measured by MCP1 and TNFα. The resident macrophage number did not increase, as reflected by unchanged F4/80 positive macrophages. However, increased macrophage activity likely resulted in increased MCP-1 and TNFα expression in response to maternal PM_2.5_ exposure. As a consequence, collagen deposition was increased in F-PM mice. Interestingly, removing PM_2.5_ in dams from conception normalised stellate cell activation markers and collagen deposition, highlighting the critical role of an optimal intrauterine environment for health outcomes.

Enhanced local inflammation and ER stress lead to NAFLD [5,55], both of which were apparent in the F-PM offspring. Consistently, recent human studies also show an increased risk of metabolic fatty liver disease from PM_2.5_ exposure [15,16]. The ER stress marker XBP1s was increased by maternal PM_2.5_ exposure in female offspring, which is also linked to antioxidants [23,56] and genes involved in lipogenesis [55,56]. Removal of PM_2.5_ in dams at conception did not prevent inflammation and ER stress in the F-Pre offspring’s liver, nor the lipid accumulation, suggesting that moving to a clean environment after conception may not be sufficient to protect lipid homeostasis in the offspring. Future studies should investigate the role of epigenetic regulation in eggs that may explain the phenomenon.

While the three mechanisms in the development of metabolic disorders, namely inflammation, ER stress, and oxidative stress [2,5,8] were all activated in F-PM offspring, their male littermates were somewhat resistant to the effects, showing relatively normal metabolic profiles. This reflects sexual dimorphism in the physiological responses to the same in utero stimulus. However, the metabolic disorders in M-Pre group (maternal PM_2.5_ removal during pregnancy) were similar to their female littermates (F-Pre), yet driven by different metabolic regulators. Proteomic analysis showed a reduced level of proteins involved in glycogen metabolism, which may result in glucose remaining in the blood. PM_2.5_ removal during pregnancy may over-correct glycogen metabolism resulting in hyperglycaemia and an over-storage of glycogen in the M-Pre offspring. In addition, several lipid metabolic markers are reduced in the M-Pre’s liver (e.g., Cyp1a2, Aldh3a2, Ugt2b1, Ces1, Cyp2f2), in line with increased liver triglyceride accumulation. This suggests that only moving to areas with relatively clean air may not be sufficient to rescue the metabolic disorder in males. Future studies can follow up on the molecular mechanisms leading to such changes in response to maternal PM_2.5_ exposure.

The sexual differences in the baseline markers of inflammation, oxidative stress, ER stress, and nutrient metabolic markers in the liver are of great interest and have rarely been reported. Most sexual dimorphism theories on physiological responses are driven by oestrogen, leading to the general perception that females are more protected than males in several organs, including the brain, lung and kidney [57,58]. Indeed, higher foetal brain testosterone has been suggested to increase the risk of autism in boys with in utero PM_2.5_ exposure [58]. Oxidative stress and systemic inflammation induced by maternal PM_2.5_ exposure during pregnancy, especially in the first trimester, have also been proposed to be involved in such a phenotype [58]. However, they were based on studies using males only [59]. This study has the advantage of examining both sexes, which demonstrates that baseline inflammation and oxidative stress profiles cannot predict the response to maternal PM_2.5_ exposure. Here, control males have higher inflammation, ER stress, and oxidative stress markers and lower levels of endogenous antioxidants in the liver than their female littermates. Males also had a lower abundance of most nutrient metabolic regulators than females. This is consistent with the higher risk of chronic liver disorders and cirrhosis in men than women [60]. However, the lower susceptibility in males in response to maternal PM_2.5_ exposure is against our initial hypothesis drawn from maternal smoking and the population risk of liver disorders due to lifestyle or environmental toxins [60]. Our previous studies on maternal smoking showed no sex differences in metabolic disorders, including glucose intolerance and liver steatosis and fibrosis [31,33,35]. Recent studies on the impact of prenatal PM_2.5_ exposure on the offspring have also demonstrated a high risk of liver disorders in male offspring at 32 weeks [26]; however, no female was included in that study for comparison. This highlights the need to study both sexes and for longer-term follow-up. The discrepancy between our study and the literature may be due to PM_2.5_ dose differences, where we used a low level that is often considered “safe” [34]. In addition, M-PM mice showed increased fasting glucose levels and a trend of increase in liver glycogen storage (PAS staining). An increase in glycogen storage may be due to a 20% reduction in Stbd1 abundance, which is involved in glycogen elimination, whereas glycogen is readily converted to glucose during fasting, which may explain increased fasting glucose levels in M-PM offspring. Inflammation (reflected by TNFa) and oxidative stress (reflected by iNOS) were suppressed in M-PM, which were unexpected. Here, there was a marginal increase in the endogenous antioxidant MnSOD and Nudt7 (involved in eliminating oxidised produce) in M-PM offspring compared with M-control mice. Albeit a lack of statistical significance, they may still have physiological significance. For example, suppressing inflammatory and oxidative stress responses in M-PM’s liver. The data in this paper are unable to fully elucidate the exact mechanism underlying the sexual difference in response to maternal PM exposure, which requires further investigation into perhaps X and Y chromosome-linked unknown genes.

## 5. Conclusions

There is a strong sex difference in liver responses to maternal PM_2.5_ exposure, with females being more susceptible than their male littermates to liver lipid deposition and oxidative stress. PM_2.5_ removal during pregnancy does not completely diminish the adverse impacts on liver health. The diminished impact of PM_2.5_ exposure in male offspring was unexpected. Future studies need to investigate this sexual dimorphism in liver responses to particulate matter exposure.

## Figures and Tables

**Table 1:** Primer and Probe Sequences for rt-PCR.

**Gene**	**NCBI Reference**	**Probe Sequence (5′ → 3′)**	**Assay ID**
iNOS	NM_010927.3	GGCCTTGTGTCAGCCCTCAGAGTAC	Mm00440502_m1
ACTA2	NM_007392.3	TAGCCCTGGCCTAGCAACACTGATT	Mm00725412_s1
COLIα1	NM_007742.3	AGTCTACATGTCTAGGGTCTAGACA	Mm00801666_g1
TNFα	NM_013693.2	CCCTCACACTCAGATCATCTTCTCA	Mm00443259_g1
18s	X03205.1	ACCGCAGCTAGGAATAATGGA	4319413E
**Gene**	**SYBR-Labelled Primer Sequence**		
MCP1	Forward primer: 5′-GTTGTTCACAGTTGCTGCCT-3′ Reverse primer: 5′-CTCTGTCATACTGGTCACTTCTAC-3′		
NOX4	Forward primer: 5′-CTGGTCTGACGGGTGTCTGCATGGTG-3′ Reverse Primer: 5′-CTCCGCACAATAAAGGCACAAAGGTCCAG-3′		

ACTA2: α-smooth muscle actin 2, COLIα1: collagen Iα1, iNOS: inducible nitric oxide synthase, MCP1: monocyte chemoattractant protein 1, NOX4: NADPH oxidase 4, TNFα: tumour necrosis factor α, 18s: 18s ribosomal RNA.

**Table 2:** Endpoint Measurements in the Offspring.

13 Weeks	Female			Male		
	Control	PM	Pre	Control	PM	Pre
**Body weight (g)**	21.7 ± 0.51	20.1 ± 0.52 *	20.8 ± 0.18	25.7 ± 0.37	26.6 ± 0.55	26.3 ± 0.44
**Liver (g)**	1.00 ± 0.06	0.94 ± 0.03	0.94 ± 0.03	1.24 ± 0.02	1.23 ± 0.03	1.11 ± 0.03 **
**Liver %**	4.58 ± 0.22	4.68 ± 0.07	4.51 ± 0.16	4.83 ± 0.07	4.63 ± 0.11	4.24 ± 0.10 **^,††^
**Liver triglyceride (μg/mg)**	84 ± 5	101 ± 6 *	107 ± 5 **	72 ± 4	66 ± 5	93 ± 8 **^,††^
**Serum triglyceride (mg/dl)**	121 ± 11	105 ± 6	97 ± 6 *	146 ± 8	138 ± 6	121 ± 6 *
**Liver PAS positive area (%) ^θ^**	64.2 ± 2.26	74.4 ± 1.82 *	71.0 ± 0.95	62.9 ± 2.23	69.1 ± 5.25	73.8 ± 2.57 **
**AUC (mM•min)**	1060 ± 17	1147 ± 30 *	1156 ± 26 *	1380 ± 37	1404 ± 67	1604 ± 27 **^,††^
**Fasting glucose (mM)**	8.57 ± 0.27	7.83 ± 0.29	7.16 ± 0.23 **	8.05 ± 0.27	8.88 ± 0.31 *	9.54 ± 0.25 **
**Fasting insulin (ng/mL)**	0.317 ± 0.041	0.225 ± 0.016 *	0.255 ± 0.027	0.278 ± 0.022	0.277 ± 0.022	0.271 ± 0.022
**HOMA**	2.95 ± 0.27	1.83 ± 0.19 **	1.98 ± 0.14 **	2.38 ± 0.23	2.50 ± 0.17	2.67 ± 0.18

Results are expressed as mean ± standard error the mean, n = 10 (^θ^ = 4–5). * *p* < 0.05, ** *p* < 0.01 vs. same sex Control; ^††^ *p* < 0.01 vs. PM. AUC: area under the curve of glucose change during glucose tolerance test; PM: offspring from dams continuously exposed to PM_2.5_; Pre: offspring from dams exposed to PM_2.5_ during pre-gestational period only.

**Table 3:** Peptide abundance area for top-3 peptides used for quantification by Peaks Studio XPro. Fold changes are compared to the Female-Control.

Protein (Abundance Unit)	Female Area			Male Area			Fold Change	Function
	F-Control	F-PM	F-Pre	M-Control	M-PM	M-Pre		
Ddah1 (×10^7^)	5.62 ± 0.20	6.89 ± 0.05 *	6.10 ± 0.13	2.64 ± 0.15	2.68 ± 0.23	3.81 ± 0.47 *	1.00:1.15:1.09: 0.47:0.48:0.68	Nitric oxide generation
Oat (×10^8^)	1.51 ± 0.09	1.59 ± 0.10	1.89 ± 0.20	0.840 ± 0.027	1.18 ± 0.31 *	0.703 ± 0.091 ##	1.00:1.05:1.24: 0.55:0.78:0.46	Mitochondrial structural protein
Cbr1 (×10^8^)	1.21 ± 0.06	1.25 ± 0.14	0.910 ± 0.049 *	0.434 ± 0.025	0.339 ± 0.021 *	0.463 ± 0.021 ##	1.00:1.04:0.83: 0.36:0.31:0.41	NADPH-dependent reductase
Cyp1a2 (×10^7^)	8.18 ± 0.67	8.31 ± 1.17	7.17 ± 0.78	22.9 ± 1.27	21.4 ± 2.19	10.3 ± 2.66 **#	1.00:1.16:0.96: 2.47:2.02:1.06	Oxidising structurally unrelated compounds, involved in drug metabolism and synthesis of cholesterol, steroids and other lipids
Rgn (×10^9^)	1.60 ± 0.11	1.37 ± 0.09	1.27 ± 0.14	0.901 ± 0.127	0.606 ± 0.044 *	0.788 ± 0.089	1.00:0.94:0.79: 0.56:0.43:0.49	Catalysing a key step in ascorbic acid (vitamin C) biosynthesis
Aldh3a2 (×10^8^)	1.14 ± 0.05	1.14 ± 0.10	1.03 ± 0.04	0.699 ± 0.066	0.628 ± 0.030	0.440 ± 0.065 ##	1.00:1.00:0.90: 0.61:0.55:0.39	Catalysing the oxidation of long-chain aliphatic aldehydes to fatty acids
Got1 (×10^8^)	2.72 ± 0.07	2.52 ± 0.09	2.47 ± 0.15	0.884 ± 0.062	0.737 ± 0.061	0.566 ± 0.067 ##	1.00:0.93:0.91: 0.33:0.27:0.24	Hepatic glyconeogensis
Stbd1 (×10^6^)	2.37 ± 0.07	1.94 ± 0.13 *	1.75 ± 0.20 *	5.00 ± 0.30	3.97 ± 0.21	2.66 ± 0.34 **#	1.00:0.88:0.74: 2.11:1.67:1.12	Elimination glycogen
Nudt7 (×10^8^)	8.65 ± 0.92	8.88 ± 0.68	7.87 ± 0.36	2.07 ± 0.17	2.39 ± 0.10	1.78 ± 0.17 #	1.00:1.03:0.91: 2.39:2.76:2.06	Eliminate oxidised CoA from peroxisomes
Ugt2b1 (×10^7^)	8.64 ± 0.55	10.1 ± 0.78	8.27 ± 0.51	19.8 ± 1.60	19.4 ± 1.87	11.8 ± 2.42 *#	1.00:1.07:0.96: 2.29:2.24:1.36	Detoxification and fatty acid metabolism
Ces1 (×10^7^)	6.95 ± 0.42	5.53 ± 0.24 *	6.20 ± 0.18	82.1 ± 3.78	74.2 ± 6.26	45.0 ± 9.48 **#	1.00:0.84:0.89: 0.56:0.43:0.44	Detoxification, lipid and carbohydrate metabolism
Ces3a (×10^8^)	3.76 ± 0.19	3.47 ± 0.51	3.41 ± 0.32	8.21 ± 0.38	7.42 ± 0.63	4.50 ± 0.95 **#	1.00:0.92:0.91: 2.18:1.97:1.19	Detoxification
Csad (×10^7^)	1.52 ± 0.210	1.26 ± 0.283	0.942 ± 0.144	6.17 ± 0.592	6.00 ± 0.876	3.50 ± 0.652 *#	1:0.83:0.62: 4.07:3.95:2.30	Taurin synthesis
Cyp2f2 (×10^7^)	8.18 ± 0.67	8.31 ± 1.17	7.17 ± 0.78	22.9 ± 1.27	21.4 ± 2.18	10.3 ± 2.66 **#	1.00:1.02:0.88: 2.80:2.62:1.26	Detox and lipase family
Sult1a1 (×10^7^)	6.65 ± 2.67	7.42 ± 3.97	7.71 ± 1.20 *	3.13 ± 1.86	2.76 ± 1.52	2.86 ± 3.39	1.00:0.90:0.76: 0.22:0.18:0.37	Cancer risk, inactivating steroid hormones and neurotransmitters, detoxification
Iigp1 (×10^7^)	3.11 ± 0.09	2.30 ± 0.36 *	2.72 ± 0.28	1.99 ± 0.18	1.95 ± 0.25	0.96 ± 0.22 *#	1.00:0.74:0.87: 0.64:0.63:0.31	Resistance to intracellular pathogens

Results are expressed as mean ± SEM, *n* = 5–6. * *p* < 0.05, ** *p* < 0.01 vs. Control of the same sex. # *p* < 0.05, ## *p* < 0.01 vs. PM of the same sex. PM: offspring from dams continuously exposed to PM_2.5_; Pre: offspring from dams exposed to PM_2.5_ during pre-gestational period only.

## Data Availability

The original data of the Western blotting have been provided to the publisher. Other data are available upon request.

## Supplementary Materials

The following supporting information can be downloaded at: https://www.mdpi.com/article/10.3390/antiox11112255/s1, Table S1: Peptide abundance area for top-3 peptides used for quantification by Peaks Studio XPro. Fold changes are compared to the Female-Control. Table S2: Functional Enrichment pathways.
