# Supplementary material for: Sex-Dependent Responses to Maternal Exposure to PM2.5 in the Offspring
Source: Antioxidants (Basel). 2022 Nov 15;11(11):2255. doi: 10.3390/antiox11112255 (PMC9686974; doi:10.3390/antiox11112255)
Supplement: Supplementary file 1 [file antioxidants-11-02255-s001.zip › antioxidants-1994390-supplementary.pdf]

Sex-dependent responses to maternal exposure to PM2.5 in the offspring

Hui Chen[1], David Van Reyk[1], Annabel Oliveira[1], Yik Lung Chan[1,2], Stephanie EL Town [1], Benjamin Rayner[3], Carol A Pollock[4], Sonia Saad[4], Jacob George[5], Matthew P Padula [1] and Brian G Oliver [1,2*]

1. School of Life Sciences, Faculty of Science, University of Technology Sydney, NSW 2007, Australia
2. Respiratory Cellular and Molecular Biology, Woolcock Institute of Medical Research, The University of Sydney, NSW 2037, Australia
3. Children's Cancer Institute, UNSW Sydney, NSW 2052, Australia
4. Kolling Institute of Medical Research, Royal North Shore Hospital, The University of Sydney, Sydney, NSW 2065, Australia
5. Storr Liver Centre, The Westmead Institute for Medical Research, Westmead Hospital and The University of Sydney, NSW 2037, Australia

Correspondence: Distinguished Professor Brian G Oliver, School of Life Sciences, Faculty of Science, University of Technology Sydney, NSW 2007, Australia; Respiratory Cellular and Molecular Biology, Woolcock Institute of Medical Research, The University of Sydney, NSW 2037, Australia. Ph: +61 2 9514 0368.Email: Brian.oliver@uts.edu.au

Materials and Method

LC/MS/MS.

Protein and metabolic pathways were examined using proteomic techniques. Samples (5 µL) were loaded onto a nanoEase Symmetry C18 trapping column (180 m x 20 mm) (3 mins/ 15 L/min) via an Acquity M-class nanoLC system (Waters, USA), and then washed onto a PicoFrit column (75 mID x 350 mm; New Objective, Woburn, MA) packed with SP-120-1.7-ODS-BIO resin. Eluted peptides were loaded into the source of a Q Exactive Plus mass-spectrometer (Thermo Scientific) using 5-30% MS buffer B (98% Acetonitrile + 0.2% Formic Acid) - 90 mins, 30-80% MS buffer B - 3 mins, 80% MS buffer B (2 mins), 80-5% for 3 min. Eluted peptides were ionised at 2400V. A data-dependent MS/MS (dd-MS2) experiment was carried out using a survey scan of 350-1500 Da at 70,000 resolution for peptides of charge state 2+ or greater with an AGC target of 3e6 and maximum Injection Time of 50ms. Peptides (top 12) were fragmented in the HCD cell using an isolation window of 1.4 m/z, an AGC target of 1e5 and a maximum injection time of 100ms. These were analysed in the Orbitrap analyser at 17,500 resolution; production fragment masses were measured (mass-range of 120 - 2000 Da). The mass of the precursor peptide was then excluded for 30 seconds.

MS/MS datafiles analysed using Peaks Studio X Pro (mouse proteome from UniProt) with a database of common contaminants with the following parameter settings. Fixed modifications: none. Variable modifications: propionamide, oxidised methionine, deamidated asparagine. Enzyme: semi-trypsin. The number of allowed missed cleavages: 3. Peptide mass tolerance: 10 ppm. MS/MS mass tolerance: 0.05 Da. Searchers were refined to include peptides with a –log10P score characterised by a False Discovery Rate (FDR) of <1%. The score is that where decoy database search matches were <1% of the total matches. Label-Free Quantification (LFQ) was performed using the PEAKS Q module.

The protein's gene name and fold change (Supplementary Table 1) were submitted to StringDB (https://string-db.org/) for Functional Enrichment using the "Proteins with Ranks/Values" Search. The functional categories enriched are listed in Supplementary Table 2.

Supplementary Table S1. Peptide abundance area for top-3 peptides used for quantification by Peaks Studio XPro. Fold changes are compared to the F-Control.

Protein (abundance unit)	Female Area			Male Area			Fold Change
	F-Control	F-PM	F-Pre	M-Control	M-PM	M-Pre	
Gstp1 (x10^8)	1.36±0.057	1.52±0.195	1.04±0.076	17.9±1.37	17.0±2.31	19.6±1.68	1:1.13:0.81: 12.61:11.84:15.21
Selenbp2 (x10^6)	4.80±1.00	3.22±1.33	1.46±0.144	55.9±4.46	51.8±3.91	72.4±11.8	1:0.67:0.30: 11.65:10.80:15.07
Mat1a (x10^8)	10.5±0.343	9.96±0.350	10.1±0.330	4.61±0.160	3.88±0.143	4.44±0.235	1:0.95:0.96: 0.45:0.38:0.44
Keg1 (x10^7)	5.26±0.348	4.44±0.489	4.62±0.315	13.6±0.281	15.6±0.464	12.0±1.56	1:0.84:0.83: 2.84:3.22:2.43
Cyp2d9 (x10^7)	1.60±0.078	1.83±0.272	1.36±0.878	5.80±0.310	5.02±0.152	3.61±0.431	1:1.14:0.85: 3.61:3.13:2.25
Aox3 (x10^7)	2.04±0.542	1.54±0.173	1.52±0.126	11.4±1.11	12.2±0.823	14.1±2.01	1:0.76:0.75: 5.32:5.74:6.66
Papss2 (x10^7)	6.05±0.328	5.53±0.357	6.67±0.325	2.42±0.080	2.08±0.100	1.87±0.254	1:0.94:1.18: 0.40:0.36:0.31
Sult1d1 (x10^7)	7.87±0.290	8.70±0.377	8.48±0.304	3.08±0.191	3.36±0.141	3.50±0.340	1:1.12:1.11: 0.48:0.41:0.43
Idh2 (x10^8)	2.10±0.110	2.18±0.125	2.45±0.116	1.05±0.052	1.16±0.065	1.14±0.059	1:1.10:1.22: 0.54:0.57:0.54
Glyat (x10^8)	1.58±0.025	1.68±0.068	1.71±0.083	2.48±0.071	2.82±0.126	2.25±0.182	1:1.12:1.05: 1.80:2.12:1.56
Sult2a8 (x10^7)	3.58±0.407	3.73±0.352	4.24±0349	13.4±0.840	14.7±1.06	14.5±1.62	1:1.05:1.20: 3.61:4.01:3.75
Ttpa (x10^7)	4.64±0.229	4.02±0.244	4.70±0.179	2.29±0.146	2.20±0.179	2.08±0.128	1:0.87:1.01: 0.49:0.47:0.44
Ugdh (x10^7)	8.88±0.351	8.59±0.435	8.63±0.360	15.7±0.681	18.0±1.12	13.1±1.84	1:0.98:0.99: 1.79:2.05:1.47
Upb1 (x10^6)	7.04±0.385	7.21±0.088	8.08±0.709	16.7±0.918	14.7±0.611	15.2±1.75	1:1.02:1.15: 2.38:2.09:2.16
Kyat3 (x10^7)	10.8±0.605	9.53±1.43	11.3±1.07	5.71±0.231	4.96±0.327	3.28±0.813	1:0.92:1.15: 0.51:0.43:0.29
Mug1 (x10^7)	2.95±0.193	2.84±0.397	2.71±0.291	6.87±0.403	7.01±0.560	5.27±0.477	1:0.96:0.92: 2.32:2.38:1.79
Gstk1 (x10^8)	3.03±0.081	3.18±0.048	3.31±0.169	2.02±0.076	2.14±0.060	1.80±0.188	1:1.03:1.11: 0.66:0.69:0.52
Ugt1a1 (x10^8)	1.21±0.047	1.12±0.039	1.12±0.035	0.788±0.105	0.655±0.004	0.517±0.064	1:0.96:0.96: 0.63:0.53:0.43
Dpys (x10^8)	2.54±0.094	2.52±0.181	2.84±0.124	1.76±0.060	1.80±0.060	1.46±0.085	1:1.04:1.23: 0.70:0.73:0.54
Serpina1c (x10^8)	1.58±0.223	1.43±0.235	1.14±0.097	3.17±0.228	2.72±0.309	4.41±0.891	1:0.88:0.72: 2.10:1.74:2.83
Ces3b (x10^7)	6.79±0.523	6.34±0.974	5.60±0.528	12.5±0.358	11.2±0.856	7.90±1.29	1:0.93:0.83: 1.84:1.65:1.17
Arhgdib (x10^6)	3.03±0.118	3.13±0.239	3.16±0.196	2.03±0.176	1.55±0.132	2.06±0.087	1:1.03:1.04: 0.67:0.51:0.68
Serpina1b (x10^7)	2.54±0.152	2.95±0.203	2.59±0.249	4.41±0.348	4.20±0.227	5.21±0.642	1:1.16:1.02: 1.74:1.66:2.05
Cyp2c29 (x10^8)	1.97±0.188	1.64±0.159	1.44±0.114	3.90±0.415	3.02±0.177	2.25±0.427	1:0.81:0.71: 1.94:1.50:1.09
Hmox2 (x10^5)	10.4±0.757	8.43±0.124	6.59±0.303	10.9±0.604	9.84±0.869	14.9±2.10	1:0.81:0.63: 1.04:0.94:1.43
Hacl1 (x10^7)	2.93±0.204	2.85±0.280	3.50±0.264	1.44±0.184	1.63±0.219	1.56±0.241	1:0.98:1.19: 0.49:0.56:0.54

Gbp9 (x10^{6})	3.38±0.39	3.35±0.244	3.55±0.172	2.31±0.165	2.15±0.137	1.67±0.262	1:0.99:1.05: 0.68:0.64:0.49

Results are expressed as mean ± SEM, n = 6. PM: offspring from dams continuously exposed to $PM_{2.5}$; Pre: offspring from dams exposed to $PM_{2.5}$ during pre-gestational period only.

Supplementary Table S2. Functional Enrichment pathways

Physiological Functions	Observed Gene Count	Background Gene Count	Strength	Matching Proteins In The Network
**Oxoacid metabolic process**	21	800	1.14	Cyp2f2,Cyp2c29,Hacl1,Dpys,Csad,Papss2,Got1,Ddah1,Ugdh,Ces1d,Cyp1a2,Ccbl2,Glyat,Mat1a,Upb1,Ugt1a1,Aldh3a2,Oat,Cyp2d9,Idh2,Gstp1
**Small molecule metabolic process**	25	1450	0.96	Cyp2f2,Cyp2c29,Hacl1,Dpys,Csad,Papss2,Got1,Ddah1,Ugdh,Ces1d,Cyp1a2,Ccbl2,Glyat,Mat1a,Upb1,Aox3,Cbr1,Ugt1a1,Nudt7,Aldh3a2,Oat,Cyp2d9,Ttpa,Idh2,Gstp1
**Carboxylic acid metabolic process**	20	754	1.14	Cyp2f2,Cyp2c29,Hacl1,Dpys,Csad,Got1,Ddah1,Ugdh,Ces1d,Cyp1a2,Ccbl2,Glyat,Mat1a,Upb1,Ugt1a1,Aldh3a2,Oat,Cyp2d9,Idh2,Gstp1
**Sulfur compound metabolic process**	11	280	1.31	Csad,Papss2,Ugdh,Gstk1,Ces1d,Glyat,Mat1a,Nudt7,Sult1a1,Sult1d1,Gstp1
**Catabolic process**	20	1680	0.8	Cyp2f2,Cyp2c29,Hmox2,Hacl1,Dpys,Csad,Got1,Ddah1,Ces1d,Cyp1a2,Mat1a,Upb1,Aox3,Stbd1,Ugt1a1,Nudt7,Oat,Cyp2d9,Ces3b,Ces3a
**Drug metabolic process**	7	52	1.85	Cyp2f2,Cyp2c29,Cyp1a2,Aox3,Cbr1,Cyp2d9,Sult1a1
**Cellular catabolic process**	18	1445	0.82	Cyp2f2,Cyp2c29,Hmox2,Hacl1,Dpys,Csad,Got1,Ddah1,Ces1d,Cyp1a2,Mat1a,Upb1,Aox3,Stbd1,Ugt1a1,Nudt7,Oat,Cyp2d9
**Organic substance catabolic process**	17	1425	0.8	Hmox2,Hacl1,Dpys,Csad,Got1,Ddah1,Ces1d,Cyp1a2,Mat1a,Upb1,Aox3,Stbd1,Ugt1a1,Nudt7,Oat,Ces3b,Ces3a
**Cellular amino acid metabolic process**	9	236	1.3	Dpys,Csad,Got1,Ddah1,Ccbl2,Glyat,Mat1a,Upb1,Oat
**Monocarboxylic acid metabolic process**	11	461	1.1	Cyp2f2,Cyp2c29,Hacl1,Ces1d,Cyp1a2,Glyat,Ugt1a1,Aldh3a2,Cyp2d9,Idh2,Gstp1
**Oxidation-reduction process**	14	917	0.9	Cyp2f2,Cyp2c29,Hmox2,Hacl1,Ugdh,Gstk1,Cyp1a2,Aox3,Cbr1,Stbd1,Aldh3a2,Cyp2d9,Idh2,Gstp1
**Metabolic process**	33	7331	0.37	Cyp2f2,Cyp2c29,Hmox2,Hacl1,Dpys,Csad,Papss2,Got1,Ddah1,Ugdh,Gstk1,Ces1d,Cyp1a2,Ccbl2,Glyat,Mat1a,Upb1,Aox3,Cbr1,Stbd1,Ugt1a1,Nudt7,Aldh3a2,Oat,Cyp2d9,Ces3b,Ces3a,Serpina1b,Ttpa,Sult1a1,Idh2,Sult1d1,Gstp1
**Organic substance metabolic process**	32	6824	0.39	Cyp2f2,Cyp2c29,Hmox2,Hacl1,Dpys,Csad,Papss2,Got1,Ddah1,Ugdh,Gstk1,Ces1d,Cyp1a2,Ccbl2,Glyat,Mat1a,Upb1,Aox3,Stbd1,Ugt1a1,Nudt7,Aldh3a2,Oat,Cyp2d9,Ces3b,Ces3a,Serpina1b,Ttpa,Sult1a1,Idh2,Sult1d1,Gstp1
**Cellular metabolic process**	31	6445	0.4	Cyp2f2,Cyp2c29,Hmox2,Hacl1,Dpys,Csad,Papss2,Got1,Ddah1,Ugdh,Gstk1,Ces1d,Cyp1a2,Ccbl2,Glyat,Mat1a,Upb1,Aox3,Cbr1,Stbd1,Ugt1a1,Nudt7,Aldh3a2,Oat,Cyp2d9,Serpina1b,Ttpa,Sult1a1,Idh2,Sult1d1,Gstp1

**Xenobiotic metabolic process**	6	74	1.63	Cyp2f2,Cyp2c29,Ugt1a1,Cyp2d9,Sult1a1,Gstp1
**Long-chain fatty acid metabolic process**	6	95	1.52	Cyp2f2,Cyp2c29,Hacl1,Cyp1a2,Cyp2d9,Gstp1
**Alpha-amino acid metabolic process**	7	173	1.33	Csad,Got1,Ddah1,Ccbl2,Glyat,Mat1a,Oat
**Primary metabolic process**	29	6369	0.38	Cyp2f2,Cyp2c29,Hacl1,Dpys,Csad,Papss2,Got1,Ddah1,Ugdh,Ces1d,Cyp1a2,Ccbl2,Glyat,Mat1a,Upb1,Aox3,Stbd1,Ugt1a1,Nudt7,Aldh3a2,Oat,Cyp2d9,Ces3b,Ces3a,Serpina1b,Sult1a1,Idh2,Sult1d1,Gstp1
**Fatty acid metabolic process**	8	310	1.13	Cyp2f2,Cyp2c29,Hacl1,Ces1d,Cyp1a2,Aldh3a2,Cyp2d9,Gstp1
**Nucleobase-containing small molecule metabolic process**	9	440	1.03	Dpys,Papss2,Ugdh,Ces1d,Glyat,Upb1,Aox3,Nudt7,Idh2
**Carboxylic acid catabolic process**	7	206	1.25	Hacl1,Csad,Got1,Ddah1,Ces1d,Mat1a,Oat
**Small molecule catabolic process**	8	318	1.12	Hacl1,Csad,Got1,Ddah1,Ces1d,Mat1a,Upb1,Oat
**Lipid metabolic process**	12	1032	0.79	Cyp2f2,Cyp2c29,Hacl1,Ces1d,Cyp1a2,Aldh3a2,Cyp2d9,Ces3b,Ces3a,Sult1a1,Sult1d1,Gstp1
**Organic cyclic compound metabolic process**	18	2614	0.56	Cyp2f2,Hmox2,Dpys,Papss2,Ugdh,Ces1d,Cyp1a2,Ccbl2,Glyat,Upb1,Aox3,Ugt1a1,Nudt7,Oat,Ttpa,Sult1a1,Idh2,Sult1d1
**Exogenous drug catabolic process**	4	33	1.8	Cyp2f2,Cyp2c29,Cyp1a2,Cyp2d9
**Alpha-amino acid catabolic process**	5	86	1.48	Csad,Got1,Ddah1,Mat1a,Oat
**Olefinic compound metabolic process**	5	91	1.46	Cyp2f2,Cyp2c29,Cyp1a2,Cyp2d9,Gstp1
**Sulfur compound biosynthetic process**	5	92	1.46	Csad,Papss2,Ugdh,Mat1a,Gstp1
**Organic hydroxy compound metabolic process**	8	420	1	Got1,Ces1d,Cyp1a2,Aldh3a2,Ttpa,Sult1a1,Idh2,Sult1d1
**Icosanoid metabolic process**	5	99	1.42	Cyp2f2,Cyp2c29,Cyp1a2,Cyp2d9,Gstp1
**Unsaturated fatty acid metabolic process**	5	100	1.42	Cyp2f2,Cyp2c29,Cyp1a2,Cyp2d9,Gstp1
**Response to toxic substance**	6	197	1.2	Cyp2f2,Gstk1,Ces1d,Glyat,Ttpa,Gstp1
**Small molecule biosynthetic process**	8	468	0.95	Csad,Got1,Ugdh,Ces1d,Upb1,Nudt7,Oat,Gstp1
**Organic cyclic compound catabolic process**	7	327	1.05	Hmox2,Dpys,Cyp1a2,Upb1,Aox3,Ugt1a1,Nudt7
**Cellular aromatic compound metabolic process**	16	2412	0.54	Cyp2f2,Hmox2,Dpys,Papss2,Ugdh,Ces1d,Cyp1a2,Ccbl2,Glyat,Upb1,Aox3,Ugt1a1,Nudt7,Sult1a1,Idh2,Sult1d1
**Arachidonic acid metabolic process**	4	53	1.6	Cyp2f2,Cyp2c29,Cyp1a2,Cyp2d9

**Organonitrogen compound metabolic process**	22	4475	0.41	Hmox2,Dpys,Csad,Papss2,Got1,Ddah1,Ugdh,Gstk1,Ces1d,Cyp1a2,Ccbl2,Glyat,Mat1a,Upb1,Aox3,Nudt7,Oat,Serpina1b,Sult1a1,Idh2,Sult1d1,Gstp1
**2-oxoglutarate metabolic process**	3	16	1.99	Got1,Ccbl2,Idh2
**Biosynthetic process**	15	2176	0.56	Csad,Papss2,Got1,Ddah1,Ugdh,Ces1d,Cyp1a2,Ccbl2,Mat1a,Upb1,Nudt7,Oat,Serpina1b,Idh2,Gstp1
**Organic acid biosynthetic process**	6	240	1.12	Csad,Got1,Ugdh,Upb1,Oat,Gstp1
**Organonitrogen compound catabolic process**	10	909	0.76	Hmox2,Dpys,Csad,Got1,Ddah1,Mat1a,Upb1,Aox3,Nudt7,Oat
**Glutamine family amino acid catabolic process**	3	22	1.85	Got1,Ddah1,Oat
**Sulfur compound catabolic process**	3	24	1.82	Csad,Mat1a,Nudt7
**Dicarboxylic acid metabolic process**	4	89	1.37	Got1,Ccbl2,Oat,Idh2
**Ribonucleoside bisphosphate metabolic process**	4	102	1.31	Papss2,Ces1d,Glyat,Nudt7
**Purine nucleoside bisphosphate metabolic process**	4	102	1.31	Papss2,Ces1d,Glyat,Nudt7
**Sulfate assimilation**	2	4	2.42	Papss2,Sult1d1
**Cellular biosynthetic process**	13	2025	0.53	Csad,Papss2,Got1,Ddah1,Ugdh,Cyp1a2,Mat1a,Upb1,Nudt7,Oat,Serpina1b,Idh2,Gstp1
**Beta-alanine metabolic process**	2	5	2.32	Dpys,Upb1
**Carboxylic acid biosynthetic process**	5	238	1.04	Got1,Ugdh,Upb1,Oat,Gstp1
**Lipid catabolic process**	5	260	1	Hacl1,Ces1d,Cyp1a2,Ces3b,Ces3a
**Sulfur amino acid catabolic process**	2	9	2.07	Csad,Mat1a
**Arginine catabolic process**	2	9	2.07	Ddah1,Oat
**Cellular nitrogen compound catabolic process**	5	279	0.97	Hmox2,Dpys,Upb1,Aox3,Nudt7
**Nucleobase catabolic process**	2	9	2.07	Dpys,Aox3
**Heterocycle catabolic process**	5	282	0.97	Hmox2,Dpys,Upb1,Aox3,Nudt7
**Organonitrogen compound biosynthetic process**	9	1096	0.63	Csad,Papss2,Got1,Ugdh,Upb1,Oat,Serpina1b,Idh2,Gstp1
**Cellular amino acid biosynthetic process**	3	58	1.43	Got1,Upb1,Oat
**Alcohol metabolic process**	5	292	0.95	Got1,Ces1d,Cyp1a2,Aldh3a2,Idh2

**Aromatic compound catabolic process**	5	297	0.95	Hmox2,Upb1,Aox3,Ugt1a1,Nudt7
**Catalytic activity**	33	5224	0.52	Cyp2f2,Cyp2c29,Hmox2,Hacl1,Dpys,Csad,Keg1,Papss2,Got1,Ddah1,Ugdh,Gstk1,Arhgdib,Iigp1,Ces1d,Cyp1a2,Ccbl2,Glyat,Mat1a,Upb1,Aox3,Cbr1,Ugt1a1,Nudt7,Aldh3a2,Oat,Cyp2d9,Ces3b,Ces3a,Sult1a1,Idh2,Sult1d1,Gstp1
**Oxidoreductase activity**	12	710	0.95	Cyp2f2,Cyp2c29,Hmox2,Ugdh,Gstk1,Cyp1a2,Aox3,Cbr1,Aldh3a2,Cyp2d9,Idh2,Gstp1
**Small molecule binding**	18	2406	0.59	Cyp2f2,Hacl1,Dpys,Csad,Papss2,Got1,Ddah1,Ugdh,Iigp1,Ccbl2,Mat1a,Aox3,Ugt1a1,Oat,Ttpa,Sult1a1,Idh2,Gstp1
**Vitamin binding**	6	134	1.37	Hacl1,Csad,Got1,Ccbl2,Oat,Ttpa
**Methyl indole-3-acetate esterase activity**	3	8	2.29	Ces1d,Ces3b,Ces3a
**Oxidoreductase activity, acting on paired donors, with incorporation or reduction of molecular oxygen, reduced flavin or flavoprotein as one donor, and incorporation of one atom of oxygen**	4	41	1.71	Cyp2f2,Cyp2c29,Cyp1a2,Cyp2d9
**Sterol esterase activity**	3	12	2.12	Ces1d,Ces3b,Ces3a
**Steroid hydroxylase activity**	4	47	1.65	Cyp2f2,Cyp2c29,Cyp1a2,Cyp2d9
**Oxidoreductase activity, acting on ch or ch2 groups**	3	13	2.08	Cyp2c29,Cyp1a2,Aox3
**Heme binding**	5	131	1.3	Cyp2f2,Cyp2c29,Hmox2,Cyp1a2,Cyp2d9
**Pyridoxal phosphate binding**	4	52	1.61	Csad,Got1,Ccbl2,Oat
**Carboxylic acid binding**	6	218	1.16	Dpys,Got1,Ddah1,Mat1a,Ugt1a1,Gstp1
**Transaminase activity**	3	22	1.85	Got1,Ccbl2,Oat
**Iron ion binding**	5	162	1.21	Cyp2f2,Cyp2c29,Cyp1a2,Aox3,Cyp2d9
**Oxidoreductase activity, acting on paired donors, with incorporation or reduction of molecular oxygen**	5	169	1.19	Cyp2f2,Cyp2c29,Hmox2,Cyp1a2,Cyp2d9
**Triglyceride lipase activity**	3	27	1.77	Ces1d,Ces3b,Ces3a

**Ion binding**	23	5439	0.35	Cyp2f2,Cyp2c29,Hmox2,Hacl1,Dpys,Csad,Papss2,Got1,Ddah1,Iigp1,Cyp1a2,Ccbl2,Mat1a,Upb1,Aox3,Ugt1a1,Nudt7,Oat,Cyp2d9,Ttpa,Sult1a1,Idh2,Gstp1
**Aromatase activity**	3	32	1.69	Cyp2c29,Cyp1a2,Cyp2d9
**Identical protein binding**	13	1962	0.54	Hacl1,Dpys,Iigp1,Ccbl2,Mat1a,Upb1,Aox3,Ugt1a1,Serpina1c,Aldh3a2,Oat,Serpina1b,Sult1a1
**Aryl sulfotransferase activity**	2	4	2.42	Sult1a1,Sult1d1
**Caffeine oxidase activity**	2	4	2.42	Cyp2c29,Cyp1a2
**Glycine n-acyltransferase activity**	2	4	2.42	Keg1,Glyat
**Organic cyclic compound binding**	21	4858	0.36	Cyp2f2,Cyp2c29,Hmox2,Hacl1,Dpys,Csad,Papss2,Got1,Ugdh,Iigp1,Cyp1a2,Ccbl2,Mat1a,Aox3,Ugt1a1,Nudt7,Oat,Cyp2d9,Ttpa,Sult1a1,Idh2
**Anion binding**	15	2678	0.47	Hacl1,Dpys,Csad,Papss2,Got1,Ddah1,Iigp1,Ccbl2,Mat1a,Aox3,Ugt1a1,Oat,Ttpa,Sult1a1,Gstp1
**Transition metal ion binding**	9	962	0.69	Cyp2f2,Cyp2c29,Dpys,Ddah1,Cyp1a2,Upb1,Aox3,Nudt7,Cyp2d9
**Oxidoreductase activity, acting on ch-oh group of donors**	4	131	1.2	Ugdh,Cbr1,Aldh3a2,Idh2
**Carbon-carbon lyase activity**	3	48	1.52	Hacl1,Csad,Got1
**Heterocyclic compound binding**	20	4771	0.34	Cyp2f2,Cyp2c29,Hmox2,Hacl1,Dpys,Csad,Papss2,Got1,Ugdh,Iigp1,Cyp1a2,Ccbl2,Mat1a,Aox3,Nudt7,Oat,Cyp2d9,Ttpa,Sult1a1,Idh2
**Transferase activity, transferring alkyl or aryl (other than methyl) groups**	3	59	1.43	Gstk1,Mat1a,Gstp1
**Estrogen 16-alpha-hydroxylase activity**	2	10	2.02	Cyp2c29,Cyp1a2
**NAD binding**	3	62	1.4	Ugdh,Aox3,Idh2
**Amino acid binding**	3	69	1.36	Dpys,Ddah1,Mat1a
**Transferase activity**	12	2088	0.48	Keg1,Papss2,Got1,Gstk1,Ccbl2,Glyat,Mat1a,Ugt1a1,Oat,Sult1a1,Sult1d1,Gstp1
**Lyase activity**	4	179	1.07	Hacl1,Csad,Got1,Ccbl2
**Cytoplasm**	36	10283	0.26	Cyp2f2,Cyp2c29,Hmox2,Hacl1,Dpys,Csad,Keg1,Got1,Ddah1,Ugdh,Gstk1,Arhgdib,Iigp1,Ces1d,Ccbl2,Glyat,Mat1a,Upb1,Aox3,Cbr1,Stbd1,Ugt1a1,Nudt7,Serpina1c,Aldh3a2,Oat,Cyp2d9,Selenbp2,Ces3b,Ces3a,Serpina1b,Ttpa,Sult1a1,Idh2,Sult1d1,Gstp1
**Intracellular**	37	12596	0.19	Cyp2f2,Cyp2c29,Hmox2,Hacl1,Dpys,Csad,Keg1,Got1,Ddah1,Ugdh,Gstk1,Arhgdib,Iigp1,Ces1d,Cyp1a2,Ccbl2,Glyat,Mat1a,Upb1,Aox3,Cbr1,Stbd1,Ugt1a1,Nudt7,Serpina1c,Aldh3a2,Oat,Cyp2d9,Selenbp2,Ces3b,Ces3a,Serpina1b,Ttpa,Sult1a1,Idh2,Sult1d1,Gstp1

**Peroxisome**	5	142	1.27	Hacl1,Gstk1,Nudt7,Aldh3a2,Idh2
**Steroid hormone biosynthesis, and Glutathione metabolism**	10	194	1.43	Cyp2f2,Cyp2c29,Ugt2b1,Gstk1,Ces1d,Cyp1a2,Ugt1a1,Cyp2d9,Sult1d1,Gstp1
**Steroid hormone biosynthesis, and Fatty acids**	8	131	1.51	Cyp2f2,Cyp2c29,Ugt2b1,Ces1d,Cyp1a2,Ugt1a1,Cyp2d9,Sult1d1
**Mixed, incl. complement and coagulation cascades, and histidine metabolism**	5	46	1.76	Mug1,Serpina1c,Ces3b,Ces3a,Serpina1b
**Steroid hormone biosynthesis, and Fatty acids**	6	123	1.41	Cyp2f2,Cyp2c29,Ugt2b1,Cyp1a2,Ugt1a1,Cyp2d9
**Methyl indole-3-acetate esterase activity, and tonb box, conserved site**	3	5	2.5	Mug1,Ces3b,Ces3a
**Alpha-amino acid metabolic process, and beta-alanine metabolism**	5	109	1.38	Dpys,Mat1a,Upb1,Aldh3a2,Oat
**Steroid hormone biosynthesis, and Fatty acids**	5	111	1.37	Cyp2c29,Ugt2b1,Cyp1a2,Ugt1a1,Cyp2d9
**Retinol metabolism, and Cytochrome P450, E-class, group I, CYP2J-like**	4	70	1.48	Cyp2c29,Ugt2b1,Cyp1a2,Ugt1a1
**Citrate cycle (TCA cycle), and Phenylalanine, tyrosine and tryptophan biosynthesis**	3	34	1.67	Got1,Ccbl2,Idh2
**Testosterone 6-beta-hydroxylase activity, and oxidative deethylation**	2	5	2.32	Ugt2b1,Cyp1a2
**Retinol metabolism**	3	45	1.54	Ugt2b1,Cyp1a2,Ugt1a1
**Mixed, incl. folic acid-containing compound metabolic process, and s-adenosylmethionine metabolic process**	3	52	1.48	Dpys,Mat1a,Upb1

**Metabolic pathways**	23	1536	0.9	Cyp2c29,Hmox2,Dpys,Csad,Keg1,Papss2,Got1,Ugdh,Ugt2b1,Gstk1,Cyp1a2,Ccbl2,Glyat,Mat1a,Upb1,Aox3,Cbr1,Ugt1a1,Aldh3a2,Oat,Selenbp2,Idh2,Gstp1
**Chemical carcinogenesis**	10	97	1.73	Cyp2c29,Ugt2b1,Gstk1,Cyp1a2,Ccbl2,Cbr1,Sult2a8,Ugt1a1,Sult1a1,Gstp1
**Metabolism of xenobiotics by cytochrome P450**	8	70	1.78	Cyp2f2,Ugt2b1,Gstk1,Cyp1a2,Cbr1,Sult2a8,Ugt1a1,Gstp1
**Drug metabolism - cytochrome P450**	6	68	1.67	Ugt2b1,Gstk1,Cyp1a2,Aox3,Ugt1a1,Gstp1
**Drug metabolism - other enzymes**	6	89	1.55	Dpys,Ugt2b1,Ces1d,Upb1,Ugt1a1,Gstp1
**Ascorbate and aldarate metabolism**	4	27	1.89	Ugdh,Ugt2b1,Ugt1a1,Aldh3a2
**Steroid hormone biosynthesis**	5	86	1.48	Cyp2c29,Ugt2b1,Cyp1a2,Ugt1a1,Cyp2d9
**Retinol metabolism**	5	92	1.46	Cyp2c29,Ugt2b1,Cyp1a2,Aox3,Ugt1a1
**Tryptophan metabolism**	4	51	1.61	Cyp1a2,Ccbl2,Aox3,Aldh3a2
**Pantothenate and CoA biosynthesis**	3	20	1.9	Dpys,Upb1,Aldh3a2
**Phenylalanine metabolism**	3	22	1.85	Keg1,Got1,Glyat
**Peroxisome**	4	85	1.39	Hacl1,Gstk1,Nudt7,Idh2
**beta-Alanine metabolism**	3	30	1.72	Dpys,Upb1,Aldh3a2
**Pentose and glucuronate interconversions**	3	34	1.67	Ugdh,Ugt2b1,Ugt1a1
**Porphyrin and chlorophyll metabolism**	3	41	1.58	Hmox2,Ugt2b1,Ugt1a1
**Cysteine and methionine metabolism**	3	50	1.5	Got1,Ccbl2,Mat1a
**Arginine and proline metabolism**	3	53	1.47	Got1,Aldh3a2,Oat
**Sulfur metabolism**	2	11	1.98	Papss2,Selenbp2
**Glutathione metabolism**	3	69	1.36	Gstk1,Idh2,Gstp1
**Biosynthesis of amino acids**	3	77	1.31	Got1,Mat1a,Idh2
**Selenocompound metabolism**	2	17	1.79	Papss2,Ccbl2
**2-Oxocarboxylic acid metabolism**	2	20	1.72	Got1,Idh2
**Bile secretion**	3	98	1.21	Ugt2b1,Sult2a8,Ugt1a1
**Tyrosine metabolism**	2	39	1.43	Got1,Aox3
**Biological oxidations**	13	213	1.51	Cyp2f2,Cyp2c29,Papss2,Ugdh,Ugt2b1,Gstk1,Ces1d,Glyat,Mat1a,Ugt1a1,Ces3a,Sult1a1,Gstp1
**Metabolism**	24	1779	0.85	Cyp2f2,Cyp2c29,Hmox2,Hacl1,Dpys,Papss2,Got1,Ddah1,Ugdh,Ugt2b1,Gstk1,Ces1d,Glyat,Mat1a,Upb1,

				Cbr1,Ugt1a1,Aldh3a2,Oat,Ces3a,Ttpa,Sult1a1,Idh2,Gstp1
**Phase II - Conjugation of compounds**	9	95	1.7	Papss2,Ugdh,Ugt2b1,Gstk1,Glyat,Mat1a,Ugt1a1,Sult1a1,Gstp1
**Glucuronidation**	3	20	1.9	Ugdh,Ugt2b1,Ugt1a1
**Protein localization**	4	104	1.31	Hacl1,Gstk1,Nudt7,Aldh3a2
**Phase I - Functionalization of compounds**	4	112	1.27	Cyp2f2,Cyp2c29,Ces1d,Ces3a
**Alpha-oxidation of phytanate**	2	6	2.24	Hacl1,Aldh3a2
**Metapathway biotransformation**	7	139	1.42	Cyp2f2,Gstk1,Cyp1a2,Glyat,Ugt1a1,Sult1a1,Gstp1
**Glucuronidation**	3	15	2.02	Ugdh,Ugt2b1,Ugt1a1
**Estrogen metabolism**	3	13	2.08	Cyp1a2,Ugt1a1,Sult1a1
**Tryptophan metabolism**	3	44	1.55	Cyp2f2,Cyp1a2,Aldh3a2
**Irinotecan pathway**	2	10	2.02	Ces1d,Ugt1a1
**Oxidative stress and redox pathway**	3	92	1.23	Gstk1,Idh2,Gstp1
**Liver**	32	1546	1.04	Cyp2f2,Cyp2c29,Hacl1,Dpys,Csad,Keg1,Papss2,Got1,Ddah1,Ugdh,Gstk1,Mug1,Ces1d,Cyp1a2,Ccbl2,Glyat,Mat1a,Upb1,Aox3,Stbd1,Ugt1a1,Nudt7,Oat,Cyp2d9,Selenbp2,Ces3b,Ces3a,Ttpa,Sult1a1,Idh2,Sult1d1,Gstp1
**Whole body**	33	9158	0.28	Cyp2f2,Cyp2c29,Hmox2,Hacl1,Dpys,Csad,Keg1,Papss2,Got1,Ddah1,Ugdh,Gstk1,Mug1,Ces1d,Cyp1a2,Ccbl2,Glyat,Mat1a,Upb1,Aox3,Stbd1,Ugt1a1,Nudt7,Oat,Cyp2d9,Selenbp2,Ces3b,Ces3a,Ttpa,Sult1a1,Idh2,Sult1d1,Gstp1
**Peroxisome**	5	132	1.3	Hacl1,Gstk1,Nudt7,Aldh3a2,Idh2
**Cytoplasm**	25	6579	0.3	Hmox2,Hacl1,Dpys,Csad,Keg1,Got1,Ugdh,Gstk1,Arhgdib,Iigp1,Ces1d,Cyp1a2,Ccbl2,Mat1a,Upb1,Ugt1a1,Nudt7,Serpina1c,Aldh3a2,Oat,Ces3a,Ttpa,Sult1a1,Idh2,Gstp1
**Microsome**	7	139	1.42	Cyp2f2,Cyp2c29,Hmox2,Cyp1a2,Ugt1a1,Aldh3a2,Cyp2d9
**Oxidoreductase**	10	623	0.93	Cyp2f2,Cyp2c29,Hmox2,Ugdh,Cyp1a2,Aox3,Cbr1,Aldh3a2,Cyp2d9,Idh2
**Endoplasmic reticulum**	12	1099	0.76	Cyp2f2,Cyp2c29,Hmox2,Iigp1,Ces1d,Cyp1a2,Stbd1,Ugt1a1,Aldh3a2,Cyp2d9,Ces3b,Ces3a
**Transferase**	14	1805	0.61	Keg1,Papss2,Got1,Ugt2b1,Gstk1,Ccbl2,Glyat,Mat1a,Sult2a8,Ugt1a1,Oat,Sult1a1,Sult1d1,Gstp1
**Pyridoxal phosphate**	4	57	1.57	Csad,Got1,Ccbl2,Oat
**Aminotransferase**	3	19	1.92	Got1,Ccbl2,Oat
**Heme**	5	165	1.2	Cyp2f2,Cyp2c29,Hmox2,Cyp1a2,Cyp2d9
**Serine esterase**	3	34	1.67	Ces1d,Ces3b,Ces3a
**Iron**	6	362	0.94	Cyp2f2,Cyp2c29,Hmox2,Cyp1a2,Aox3,Cyp2d9
**Monooxygenase**	4	127	1.22	Cyp2f2,Cyp2c29,Cyp1a2,Cyp2d9

**Lyase**	4	159	1.12	Hacl1,Csad,Cyp1a2,Ccbl2
**Serine protease inhibitor**	3	83	1.28	Mug1,Serpina1c,Serpina1b
**Pyridoxal phosphate-dependent transferase, major domain**	4	42	1.7	Csad,Got1,Ccbl2,Oat
**Pyridoxal phosphate-dependent transferase**	4	42	1.7	Csad,Got1,Ccbl2,Oat
**Carboxylesterase type B, active site**	3	22	1.85	Ces1d,Ces3b,Ces3a
**Carboxylesterase, type B**	3	26	1.78	Ces1d,Ces3b,Ces3a
**Cytochrome P450, E-class, group I**	4	82	1.41	Cyp2f2,Cyp2c29,Cyp1a2,Cyp2d9
**Cytochrome P450, conserved site**	4	93	1.35	Cyp2f2,Cyp2c29,Cyp1a2,Cyp2d9
**Carboxylesterase type B, conserved site**	3	26	1.78	Ces1d,Ces3b,Ces3a
**Sulfotransferase domain**	3	40	1.6	Sult2a8,Sult1a1,Sult1d1
**Cytochrome P450**	4	101	1.32	Cyp2f2,Cyp2c29,Cyp1a2,Cyp2d9
**Glycine N-acyltransferase**	2	4	2.42	Keg1,Glyat
**Glycine N-acyltransferase, C-terminal**	2	4	2.42	Keg1,Glyat
**Pyridoxal phosphate-dependent transferase domain 1**	3	34	1.67	Got1,Ccbl2,Oat
**Glycine N-acyltransferase, N-terminal**	2	4	2.42	Keg1,Glyat
**Cytochrome P450 superfamily**	4	101	1.32	Cyp2f2,Cyp2c29,Cyp1a2,Cyp2d9
